# Danhong Injection Combined With t-PA Improves Thrombolytic Therapy in Focal Embolic Stroke

**DOI:** 10.3389/fphar.2018.00308

**Published:** 2018-04-06

**Authors:** Min Li, Jing Zhou, Weifeng Jin, Xiaohong Li, Yuyan Zhang

**Affiliations:** School of Life Science, Zhejiang Chinese Medical University, Hangzhou, China

**Keywords:** Danhong injection, t-PA, combination therapy, embolic, cerebral ischemia

## Abstract

**Background:** Hemorrhagic transformation, neurotoxicity, short treatment time windows, and other defects are considered as the major limitations for the thrombolytic therapy. This study is devoted to figure out whether Danhong injection (DHI) combined with tissue-plasminogen activator (t-PA) could extend the treatment time windows and ameliorate brain injury, hemorrhagic complication and BBB disruption after focal embolic stroke.

**Methods:** *In vitro*, the combined concentrations of DHI and t-PA were added to wells reacted with plasminogen and D-Val-Leu-Lys-AMC. The optimum ratio of the combination of DHI plus t-PA was explored by detecting relative fluorescent. *In vivo* experiments, we firstly investigated the optimal dose of t-PA and Danhong injection for focal embolic stroke. The neurological deficit score, infarct volume and brain edema were assessed. Secondly, we proved that the combination group extended the thrombolytic window for treatment of focal embolic stroke. The neurological deficit score, infarct volume, brain edema and hemorrhagic complication were assessed, while levels of BAX, Bcl-2 and caspase-3 in brain tissue were analyzed by real-time polymerase chain reaction. Finally, to ask whether combination therapy with DHI plus t-PA protected the blood–brain barrier in a rat model of focal embolic stroke, neurological deficit score, ELISA, RT-PCR, western blot and fluorescence were used to detect the indicators of blood–brain barrier, such as tight junction protein, blood–brain barrier permeability and related gene expression.

**Results:** *In vitro*, plasmin activity assays showed that the combination of t-PA with DHI at about 1:1.6 w/v ratio increased by almost 1.4-fold the plasmin-generating capability of t-PA. *In vivo* experiments, the results showed that the combination of Danhong injection (4 mL/kg) and t-PA (2.5 mg/kg) could extend the t-PA treatment time windows to 4.5 h. And the combination t-PA (2.5 mg/kg) with DHI (4 mL/kg) ameliorated neurological score, cerebral infarction, brain edema, brain hemorrhage, and BBB disruption.

**Conclusion:** Combination therapy with Danhong injection (4 mL/kg) plus t-PA (2.5 mg/kg) could extend the t-PA treatment time windows to 4.5 h, ameliorate BBB disruption, reduce infarction, brain swelling and hemorrhage after ischemic stroke.

## Introduction

Ischemic stroke is a leading cause of death, disability, and massive socioeconomic loss worldwide (Wang et al., 2014). By stimulating thrombolysis and rescuing the ischemic brain via reopening occluded vessels, intravenous administration of recombinant tissue plasminogen activator (t-PA) remains the most effective intervention with FDA approval for emergency treatment of stroke (Whiteley et al., 2014). However, its efficacy and safety only ensured when administered in 3 h after the onset of symptoms. Only a small percentage of patients with ischemic stroke are eligible for t-PA treatment. In addition, t-PA thrombolytic therapy is inevitable with hemorrhage, reperfusion injury and other complications. The risk of symptomatic intracranial hemorrhage, poor thrombolytic perfusion rate, neurotoxicity, and a short treatment time window comprise the major challenges of its application (Bambauer et al., 2006; Weintraub, 2006). Improving t-PA thrombolytic therapy is a high priority in stroke research (Zhu et al., 2010), and it may both lengthen the time-to-treatment window and make reperfusion therapy safer and more efficacious (Marder and Stewart, 2002; Dávalos, 2005). The pathological mechanism of ischemia stroke is complex, involving excitotoxicity, oxidative damage, inflammation and Ca^2+^ overload. The combination therapy in the treatment of acute cerebral infarction has achieved good therapeutic effect. Minocycline combined with t-PA could extend the treatment time window and decrease hemorrhagic transformation after focal embolic stroke in type 1 diabetic rats. Therefore, it is imperative to seek combination therapies that will truly extend the treatment time window, while reducing the risk of t-PA-associated hemorrhagic transformation, and enhancing thrombolytic efficacy (Whiteley et al., 2014). For this perspective, Danhong injection may be a compelling candidate.

Danhong injection (DHI), a Chinese Materia Medica standardized product extracted from *Radix Salviae miltiorrhizae* and *Flos Carthami tinctorii*, is used extensively for the treatment of cerebrovascular diseases such as acutely cerebral infarction in clinic (Du et al., 2011; He et al., 2012). *Radix Salviae miltiorrhizae* and *Flos Carthami tinctorii* are both well-known Chinese herbal medicinal formula for treated cardiovascular and cerebrovascular diseases. The main bioactive constituents of DHI are danshensu, salvianic acid A and B, protocatechuic aldehyde, rosmarinic acid and some saccharides. These compositions are the material basis of neuroprotective effect of DHI. More experimental evidences have shown that DHI exhibited diverse pharmacological effects, including protection against vascular endothelium injury (Gao et al., 2011; Zhi et al., 2012), improvement in microcirculation (Jung et al., 2011), inflammation, and neuroprotective (He et al., 2012).

Combination therapy to extend the treatment time window may have the following aspects. The combination approach of t-PA and DHI may reduce the expression of PAI-1 and the dosage of t-PA, thereby reducing the side effects of t-PA, and prolonging the treatment time window. DHI of cerebrovascular expansion in combined treatment may reduce the t-PA-induced cerebral vasoconstriction and improve the efficiency of thrombolysis. DHI could inhibit the activation of MMP-9 and protect the blood–brain barrier, which may reduce the MMP-9 activation caused by t-PA and the destruction of the blood–brain barrier. It might lengthen the treatment time windows, enhance t-PA thrombolytic efficacy and reduce the side effects of t-PA-induced. In this study, we aimed at evaluating a novel combination approach of low-dose t-PA and Danhong injecting in a rat model of focal embolic stroke, which might lengthen the treatment time windows, enhance t-PA thrombolytic efficacy, while reducing its associated complications related to intracerebral hemorrhagic transformation.

## Materials and Methods

### Plasmin Activity Assay

A 96-well plate was pre-loaded with fibrinolytic solution at 37°C for 1 h. After washing plate, 50 μL of the indicated individual or combined concentrations of Danhong injection, t-PA and BSA were added. Thereafter, a fluorogenic plasmin substrate, D-Val-Leu-Lys-AMC, was added to wells of 96-well plate in a final volume of 100 μL PBS. It was incubated at 37°C for 30 min. Plasmin generation was read on a fluorescent plate reader with excitation set at 360 nm and emission at 460 nm (Zhu et al., 2010). The fluorescence intensity in the very dilute solution was proportional to the concentration of the fluorescent substance, so the final result was represented as fold of plasmin activity generated by t-PA.

### Animal Models of Focal Embolic Cerebral Ischemia

All experiments were performed following an institutionally approved protocol in accordance with the National Institutes of Health Guide for the Care and Use of Laboratory Animals. Adult male Sprague-Dawley rats (body weight, 250–280 g) were purchased from the Animal Center of Zhejiang Chinese Medical University, Hangzhou, China (Laboratory Animal Certificate: SCXK:2014-0001). All rats were subjected to a focal embolic stroke by occluding the MCA with a fibrin-rich allogeneic clot (Fan et al., 2012). Its thrombolytic reperfusion time window and hemorrhagic transformation closely mimic the clinical situation, which has been most commonly used for thrombolytic stroke studies. Expose the common carotid artery (CCA), internal carotid artery (ICA), external carotid artery (ECA) with careful blunt dissection. Necessary prevention and care should be provided to avoid injury to the vagal nerve during dissection. Clamp the CCA and the ICA with an aneurysm clip, and apply a 4-0 silk suture loosely around the trunk of the ECA near the bifurcation. Create a partial arteriotomy on the ECA, and then insert the tip of the clot into the arteriotomy. Meanwhile, the regional cerebral blood flow was used to monitor for up to 1 h after treatment (Henninger et al., 2009). The inclusion criteria were stable 50% or less rCBF of pre-ischemic baseline for up to 1 h after embolization. It’s worth noting that rectal temperature was monitored and maintained at 37°C using a heating blanket.

### Experiments and Treatment Groups

All drug treatments and outcome assessments were performed by an investigator blinded to the surgical groups (Lapchak et al., 2013). Three sets of experiments were performed. In the first set of experiments, rats were used to test the optimal dose of t-PA and Danhong injection (DHI) in the embolic stroke animal model. Four hours after initiation of ischemia, 70 animals (*n* = 5 per group) were treated intravenously with saline, high-dose t-PA (10 mg/kg), DHI (4 mL/kg), combination of t-PA (1.25 mg/kg) plus DHI (2 mL/kg) (DHI-L + tPA-L), t-PA (2.5 mg/kg) plus DHI (4 mL/kg) (DHI-M + tPA-M), and t-PA (5 mg/kg) plus DHI (8 mL/kg) (DHI-H + tPA-H). Our initial choice of combination doses for t-PA plus DHI *in vivo* was based on plasmin generation data *in vitro*, where the ratio of combining t-PA with DHI was about 1:1.6. Brain infarction volume, brain edema and neurologic outcomes, was quantified at 24 h after stroke.

In the second set of experiments, a total of 90 rats were used to investigate whether Danhong injection could extend the t-PA treatment time windows. Rats were divided randomly into nine groups: (1) sham group; (2) vehicle group; (3) t-PA (10 mg/kg, treated at 4 h after stroke); (4) DHI (4 mL/kg, treated at 4 h after stroke); (5) DHI + tPA + 2 h (dosage: experiment 1 results, treated at 2 h after stroke); (6) DHI + tPA + 4 h (dosage: experiment 1 results, treated at 4 h after stroke); (7) DHI + tPA + 4.5 h (dosage: experiment 1 results, treated at 4.5 h after stroke); (8) DHI + tPA + 5 h (dosage: experiment 1 results, treated at 5 h after stroke); (9) DHI + tPA + 6 h (dosage: experiment 1 results, treated at 6 h after stroke). In this segment, we tested hemorrhagic transformation, neurotoxicity, infarct volumes, edema volume and expression of apoptosis-related gene.

In the third set of experiments, rats were tested to protect the blood–brain barrier in a rat model of focal embolic stroke. Four hours after initiation of ischemia, 75 animals (*n* = 5 per group) were treated intravenously with saline, t-PA (5 mg/kg), DHI (4 mL/kg), combination of t-PA (2.5 mg/kg) plus DHI (4 mL/kg). High-dose t-PA treated at 4 h after stroke caused higher 24 h mortality. To avoid unacceptable high mortality, the dose of t-PA to 5 mg/kg was adjusted. ELISA, RT-PCR, western blot and fluorescence were used to detect the indicators of blood–brain barrier, such as tight junction protein, blood–brain barrier permeability and related gene expression.

### Analysis of Neurological Score, Infarct Volumes and Edema Volume

At 24 h after ischemia, rats were assessed with a 4-point neurologic deficit scale that has been extensively used for rat models of stroke. The neurological deficit scoring system, based on the Bederson (Bederson et al., 1986) and Garcia score (Garcia et al., 1995), used a scale from 0 to 4: (0) no observable deficit; (1) decreased forelimb resistance to a lateral push; (2) forelimb flexion; (3) circling behavior in addition to the former symptoms; and (4) deficiency in spontaneous walking.

Rats were euthanized at 24 h after ischemia. The rat brain was immediately removed and placed at -20°C for 10 min. Coronal brain sections were cut at 2 mm from the frontal tips, and stained with 2% 2.3.5-triphenyltetrazolium chloride (TTC, Sigma, United States) at 37°C for 15 min in the dark (Wang et al., 2014). The infarct area in each slice was photographed via a digital camera and quantified via computer-assisted image analysis. Infarct volume was expressed by a percentage of the total volume of slices.

In paralled with infarct volume, brain edema with determined used the wet/dry method as previously described (Guo et al., 2014), in which percentage brain water = [(wet weight-dry weight)/wet weight]^∗^100%.

### Quantification of Intracerebral Hemorrhage

T-PA thrombolytics alone exhibited a trend for infarct reduction, but significantly elevated intracerebral hemorrhage. In order to study whether the treatment of combination could decrease intracerebral hemorrhage treated by t-PA alone, we measured the intracerebral hemorrhage as we described previously (Ploen et al., 2014). Unstained coronal sections were taken every 400 μm, and scored by investigators blinded to the experiment. Hemorrhage scores range from 0 to 4: 0 = no hemorrhage; 1 = single petechial hemorrhage; 2 = confluent petechial; 3 = a single space occupying parenchymal hemorrhage encompassing < 30% infarction area; and 4 = multiple space-occupying parenchymal hemorrhage > 30% of infarction area.

### Real Time Reverse Transcription Polymerase Chain Reaction (RT-PCR) Assay

Total RNA was separated from the ischemic brain by using TRLzol reagent, and detected concentration by NanoVue Plus (A260/A280). Then, total RNA subsequently was reverse-transcribed to cDNA by using TaqMan Reverse Transcription Reagents. The reaction condition was installed for 37°C for 15 min, 85°C for 5 s, 4°C at 10 min. Real time PCR was performed using SYBR Green PCR Master Mix reagent kits and the specific primers. After the reaction, the fusion curve was analyzed to identify the specificity of PCR products. Using GAPDH as an internal control, and the data were analyzed by using the comparative threshold cycle (*C*t) method.

### Measurement of MMP-9, PAI-1 and P-Selectin Levels

To determine changes of blood–brain barrier permeability and t-PA inhibition, blood plasma samples were collected at 24 h after ischemia. 10 μl of plasma sample was applied in each well and all the samples were run in duplicates. The MMP-9, PAI-1, and P-selectin levels were measured by ELISA kits according to the manufacturer’s instructions, respectively. The content of MMP-9, PAI-1, and P-selectin in plasma was calculated by establishing standard curve.

### Blood–Brain Barrier Permeability for Evans Blue

Evans blue had high affinity with plasma albumin. They could form an Evans Blue -albumin complex, which could limit Evans Blue to penetrate BBB. Therefore, Evans Blue permeability was an objective index to measure the variation of BBB permeability. To quantify postischemic blood brain barrier (BBB) permeability, a previously established fluorometric assay were used to assess extravasation of albumin-bound Evans Blue (Ploen et al., 2014). Animals were briefly reanesthetized 22 h after stroke, and 2% Evans Blue in phosphate buffered saline (4 mL/kg) was injected into the femoral vein. Two hours later, rats were transcardially perfused with saline until the outflow of liquid was limpid. Centrifuge tubes containing brain tissues were added 2 mL/g formamide, and took water bath in a light resistant container. After centrifugation centrifugal, supernatant was collected in centrifuge tubes. Evans Blue content of each hemisphere was quantified by fluorometry. The results were expressed by μg/g of brain tissue.

### Western Blotting Analysis

Rats were euthanized 24 h after ischemia. Brain tissues were homogenized in lysis buffer including protease inhibitors on ice. After centrifugation, supernatant was collected to centrifuge tubes, and total protein concentrations were determined using the NanoVue Plus. Protein samples were separated by 10% SDS-PAGE and transferred to polyvinylidene difluoride membranes. The membrane was subsequently blocked with 5% BSA in Tris-buffered saline with Tween 20 solution and incubated with polyclonal occludin (Abcam, ab167161) and MMP-2 (Abcam, ab92536) for 12 h at 4°C. Peroxidase-linked anti-rabbit IgG (Boster, BST12B08A54) and ECL reagents (Boster, 11F16B11) were used as a detection system. The expression of MMP-2 and occludin protein was analyzed by using gel imaging analysis system.

### Statistical Analysis

Data were expressed as mean ± SD. Statistical analysis was carried out by using a one-way analysis of variance as well as the least significant difference test. Differences with *P* < 0.05 were considered statistically significant.

## Results

### Danhong Injection Amplifies t-PA-Mediated Plasmin Generation *in Vitro*

**FIGURE 1:**
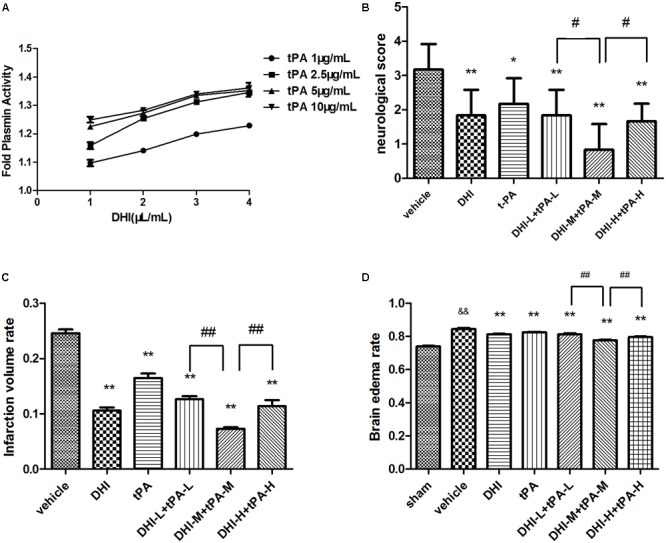
Effect of DHI on tPA-dependent plasmin generation *in vitro* and acute brain tissue outcomes of different combination therapy in stroke rats. **(A)** A range of concentrations of tPA (1, 2.5, 5, and 10 μg/mL) with or without the indicated concentrations of DHI (1, 2, 3, and 4 μL/mL) were added to wells of 96-well plate. Plasmin activity was represented as fold of plasmin activity related to 1 μg/mL of tPA alone. **(B)** At 24 h after stroke, neurological score were quantified. **(C)** Ischemic infarct volume rates were quantified at 24 h after stroke. **(D)** Brain edema was assessed at 24 h after stroke. Data were expressed as mean ± SD, *n* = 6 per group. ^∗^*P* < 0.05, ^∗∗^*P* < 0.01 vs. vehicle group. ^&&^*P* < 0.01 vs. sham group. ^#^*P* < 0.05, ^##^*P* < 0.01 vs. DHI-M+tPA-M.

*In vitro*, plasmin activity assays showed Danhong injection (4 μL/mL) combined with t-PA (2.5 μg/mL) significantly amplified t-PA-converted plasmin generation (**Figure 1A**). Equal activity of plasmin could be reached by different combinations with lower dose t-PA with DHI, such as t-PA 10 μg/mL plus DHI 4 μL/mL, t-PA 5 μg/mL plus DHI 4 μL/mL, t-PA 2.5 μg/mL plus DHI 4 μL/mL. The data of assay indicated that the plasmin-generating capability of combining t-PA with DHI at 1:1.6 w/v ratio was increased by almost 1.4-fold of t-PA *in vitro*. To explore the optimal dose of t-PA and Danhong injection, we selected appropriate dosage for animal experiment in the ratio of t-PA and DHI to 1:1.6.

### Explore the Optimal Dose of t-PA and Danhong Injection for Focal Embolic Stroke

**FIGURE 2:**
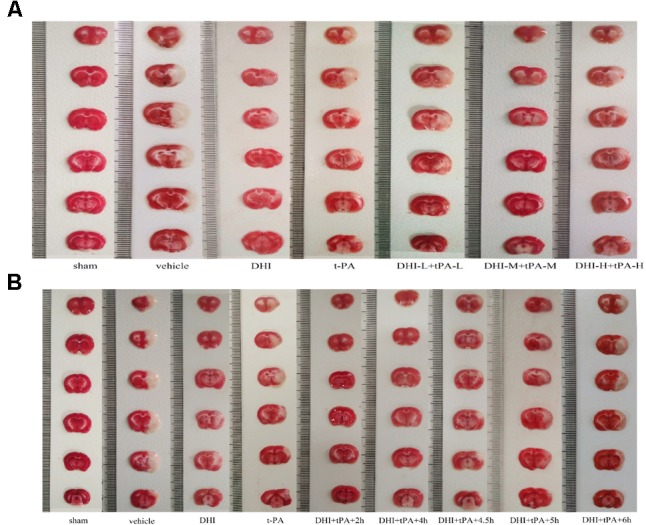
Representative images for TTC stained ischemic brain infarctions. **(A)** Effect of different combination groups on cerebral infarction volume. **(B)** Effect of treatment time window on cerebral infarction volume.

Brain infarction volume, brain edema and neurologic outcomes, were quantified at 24 h after stroke. The infarct size was observed by using vital staining with 2, 3, 5-triphenyltetrazolium chloride (TTC). Representative images for TTC stained ischemic brain infarctions were shown in **Figure 2**. Normal brain tissue was stained deep red, whereas the infarct tissue was not stained. Compared to model group, the infarct volume was significantly reduced in each treatment group. Infarction volume in the t-PA (2.5 mg/kg) plus DHI (4 mL/kg) combination group was significantly smaller than the other combination groups (**Figure 1C**). Meanwhile, the Brain edema in the model group was significantly increased in comparison to the sham group. The combination of DHI (4 mL/kg) plus t-PA (2.5 mg/kg) showed the smallest Brain edema, followed the combination of t-PA (1.25 mg/kg) plus DHI (2 mL/kg) and the combination of t-PA (5 mg/kg) plus DHI (8 mL/kg), when compared with the model group (**Figure 1D**).

Similarly, neurological score was also significantly reduced in the treatment groups. Neurological deficit score in the t-PA (2.5 mg/kg) plus DHI (4 mL/kg) combination group was significantly lower than that of the other treatment groups (**Figure 1B**). These data indicated that this combination thrombolytic therapy was more effective and specific for fibrinolysis. So, the combination of t-PA (2.5 mg/kg) plus DHI (4 mL/kg) was selected as a combined group for subsequent research.

### Low-Dose of t-PA, in Combination With Danhong Injection, Extends the Thrombolytic Window for Treatment of Focal Embolic Stroke in Rats

**FIGURE 3:**
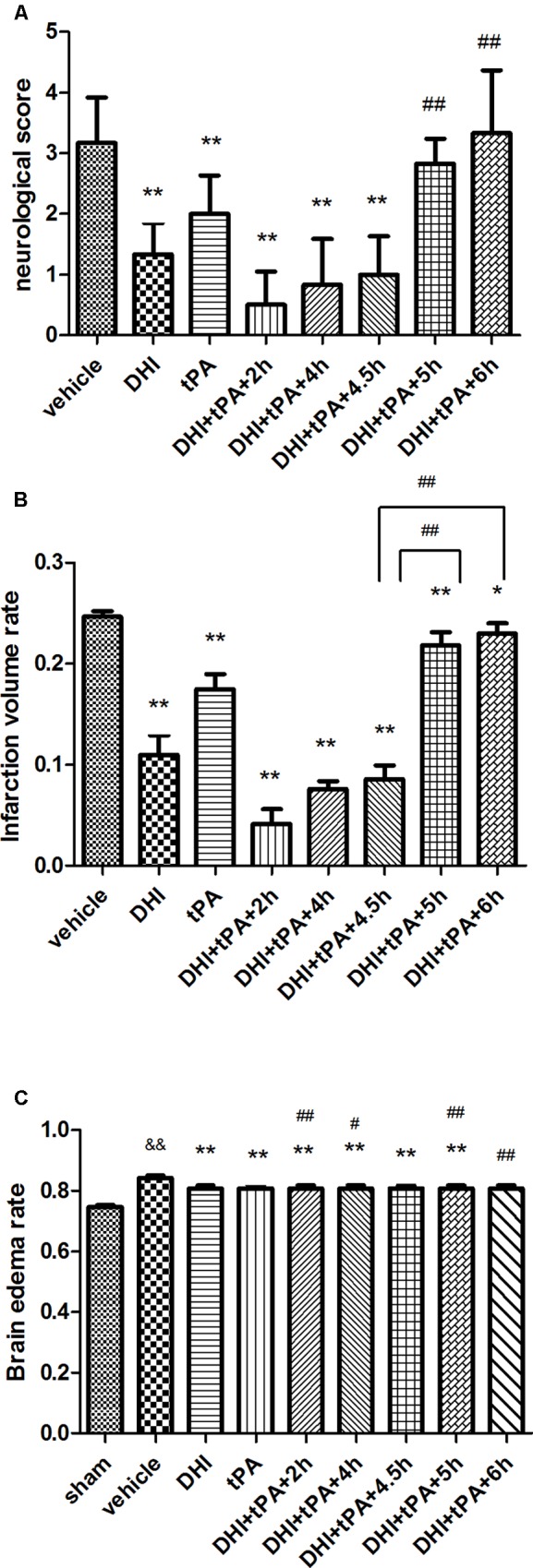
Effect of different treatment time on neurological score, cerebral infarction volume and cerebral edema. **(A)** At 24 h after stroke, neurological score were quantified. **(B)** Ischemic infarct volume rates were quantified at 24 h after stroke. **(C)** Brain edema was assessed at 24 h after stroke. Data were expressed as mean ± SD, *n* = 6 per group. ^∗^*P* < 0.05, ^∗∗^*P* < 0.01 vs. vehicle group. ^&^*P* < 0.05, ^&&^*P* < 0.01 vs. sham group. ^#^*P* < 0.05, ^##^*P* < 0.01 vs. DHI+tPA+4.5 h.

Focal stroke dramatically increased the infarct volume in the ischemic brain hemisphere. Cerebral infarct volume was decreased after the administration of medicine. The combination of t-PA plus DHI administered at 2, 4, and 4.5 h after stroke significantly lowered infarction volume compared with t-PA alone treatment. At the same time, the combined group administered at 2, 4, and 4.5 h after stroke, had the similar ability to reduce the volume of cerebral infarction, and achieved the same effect of protecting brain tissue. However, infarction volume in the combination groups treated at 5 and 6 h after stroke was significantly bigger than the other treatment groups (**Figures 2, 3B**). As the results of cerebral infarction volume, the neurological scores of the combination groups administered at 2, 4, and 4.5 h were better than the combination groups administered at 5 and 6 h after stroke (*P* < 0.01). There was no statistically significant difference between the combination therapy treated at 4.5 h after ischemia and combination therapy administered at 2 and 4 h after stroke (**Figure 3A**). It indicated that DHI combinated t-PA indeed extended the thrombolytic window to 4.5 h for treatment of focal embolic stroke in rats.

Then, the brain edema was examined in the different treatment groups (**Figure 3C**). In addition to the combination group treated at 6 h after stroke, brain edema of the other combination groups was significantly reduced as compared with the model group. Meanwhile, the rate of brain edema in the combination group treated at 5 and 6 h after stroke was significantly higher than that of the combined group administrated 4.5 h after stroke. t-PA increased macroscopic hemorrhage on unstained coronal cryosections compared with the vehicle group (mean hemorrhage score 2.3 ± 0.7 vs. 1.1 ± 0.5). The combination of t-PA plus DHI treated at 4.5 h after stroke decreased hemorrhage compared with the other combination groups.

**FIGURE 4:**
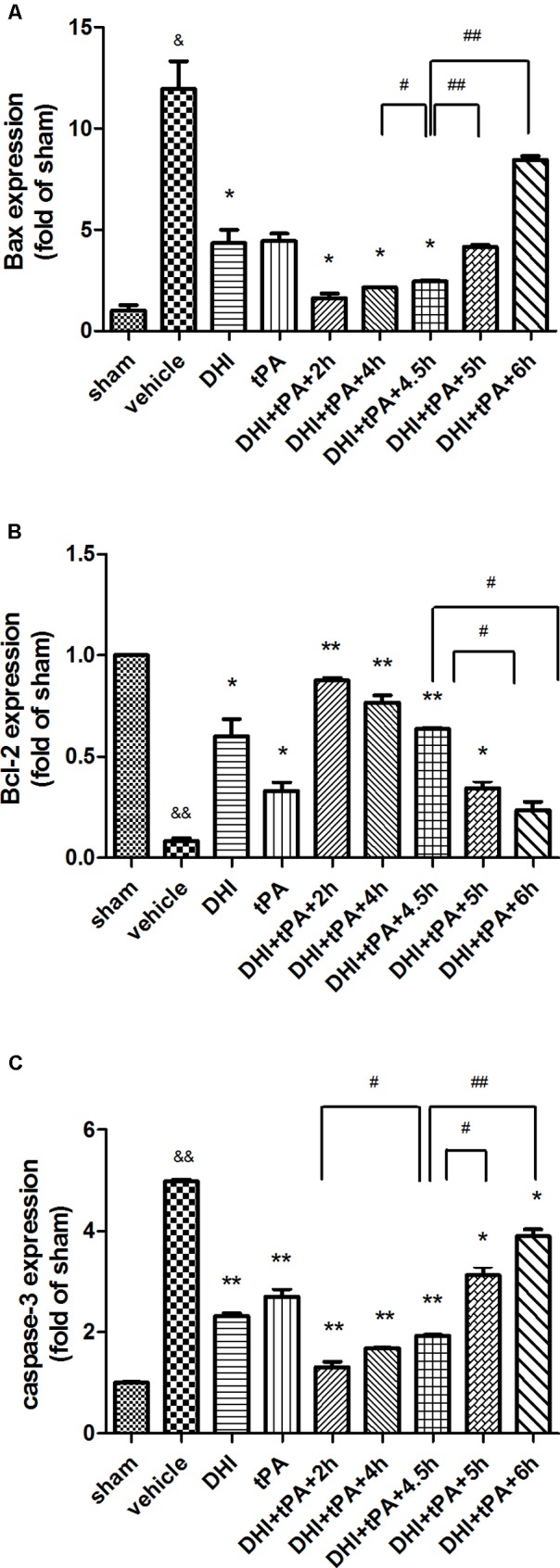
Effect of different treatment time on the mRNA transcriptions of Bax, Bcl-2, caspase-3. **(A–C)** The real time PCR was performed to determine the relative levels of Bax, Bcl-2, caspase-3 mRNA transcriptions at 24 h after stroke. Data were expressed as mean ± SD, *n* = 6 per group. ^∗^*P* < 0.05, ^∗∗^*P* < 0.01 vs. vehicle group. ^&^*P* < 0.05, ^&&^*P* < 0.01 vs. sham group. ^#^*P* < 0.05, ^##^*P* < 0.01 vs. DHI+tPA+4.5 h.

In order to determine the Danhong injection plus t-PA prolonged the thrombolytic window to 4.5 h for treatment of focal embolic stroke in rats, real time RT-PCR was performed to determine the relative levels of BAX, Bcl-2, and caspase-3 mRNA transcriptions in rat tissue. After stroke, the expression of BAX and caspase-3 mRNA was significantly upregulated. The combination group treated at 4.5 h after ischemia was significantly reduced the expression of BAX and caspase-3 mRNA, as same as the combination group treated at 2 and 4 h after stroke (**Figures 4A,C**). However, the combination group treated at 5 and 6 h after ischemia could not reduce the BAX mRNA transcription. Furthermore, compared with combination group treated at 5 and 6 h after ischemia, the Bcl-2 signals were strengthened in combination group treated at 4.5 h after stroke (**Figure 4B**). Thus, Danhong injection extended the t-PA treatment time windows to 4.5 h.

### Combination of DHI Plus t-PA Protects the Blood–Brain Barrier in Focal Embolic Stroke

**FIGURE 5:**
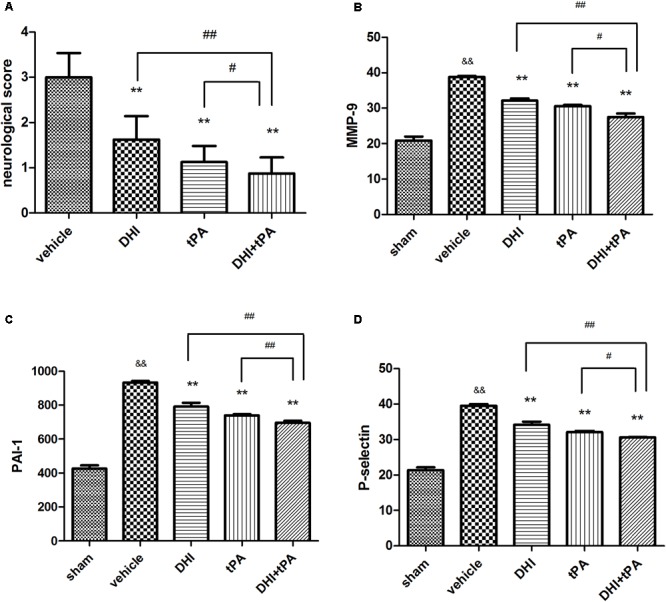
Effect of DHI combinated with tPA in neurological core and the levels of MMP-9, PAI-1, P-selectin at 4.5 h after initiation of ischemia. **(A)** At 24 h after stroke, neurological score were quantified. **(B–D)** The levels of MMP-9, PAI-1, P-selectin were tested at 24 h after stroke. Data were expressed as mean ± SD, *n* = 6 per group. ^∗∗^*P* < 0.01 vs. vehicle group. ^&&^*P* < 0.01 vs. sham group. ^#^*P* < 0.05, ^##^*P* < 0.01 vs. DHI+tPA.

**FIGURE 6:**
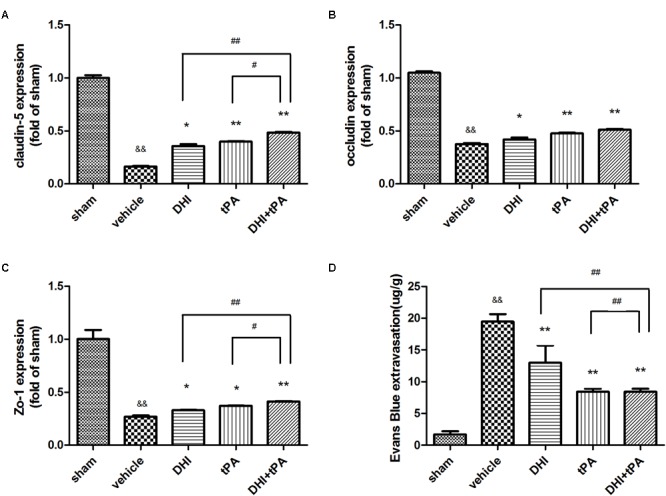
**(A–C)** The real time RT-PCR was performed to determine the relative levels of claudin-5, occludin, ZO-1 mRNA transcriptions at 24 h after stroke. **(D)** Evans Blue extravasation in the ischemic hemi sphere was quantified by fluorometry. Data were expressed as mean ± SD, *n* = 6–8 per group. ^∗^*P* < 0.05, ^∗∗^*P* < 0.01 vs. vehicle group. ^&&^*P* < 0.01 vs. sham group. ^#^*P* < 0.05, ^##^*P* < 0.01 vs. DHI+tPA.

**FIGURE 7:**
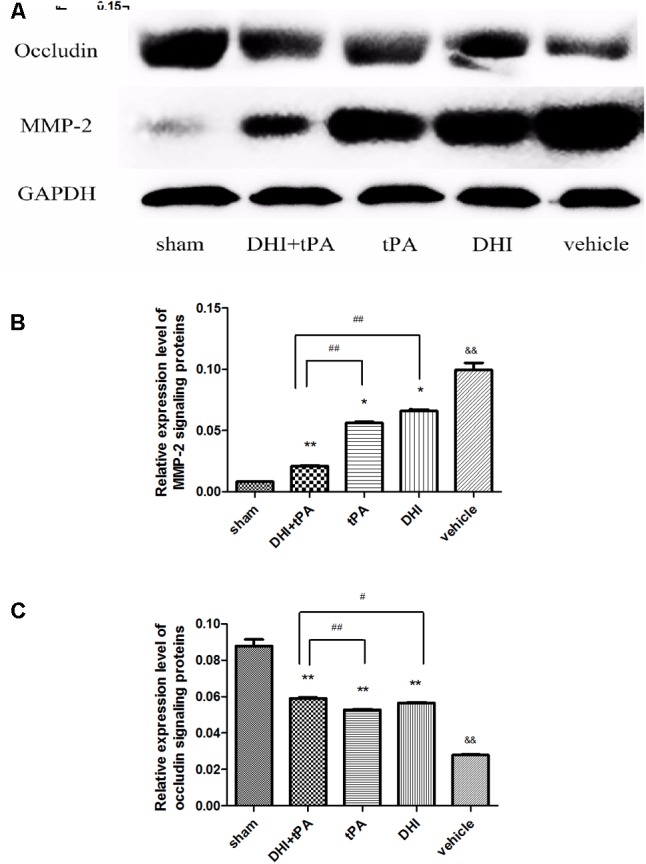
At 24 h after stroke, the expression of MMP-2 and occludin protein treated with saline, DHI, tPA, and DHI combinated with tPA. **(A)** Representative blots of the MMP-2, occludin in the five groups. **(B,C)** The relative expression level of MMP-2 and occluding signaling proteins. Data were expressed as mean ± SD, *n* = 6–8 per group. ^∗^*P* < 0.05, ^∗∗^*P* < 0.01 vs. vehicle group. ^&&^*P* < 0.01 vs. sham group. ^#^*P* < 0.05, ^##^*P* < 0.01 vs. DHI+tPA.

To increase the validity of our findings, we examined the effect that combination of DHI plus t-PA protected the blood–brain barrier. At 24 h after stroke, neurological score was significantly reduced in DHI group, t-PA group, and combined group. Neurological score in the combination of t-PA plus DHI was significantly smaller than the other treatment groups (**Figure 5A**). After stroke onset, plasma MMP-9, PAI-1, and P-selectin were significantly elevated, and the combination of t-PA plus DHI significantly suppressed the plasma MMP-9, PAI-1, P-selectin levels compared with t-PA alone treatment (**Figures 5B–D**). These data indicated that this combination thrombolytic therapy was more effective and specific for cerebral ischemic stroke. Then, RT-PCR was performed to determine the relative levels of claudin-5, occludin, ZO-1 mRNA transcriptions in rat brain tissue. The results showed weak claudin-5, occludin, ZO-1 positive signals in the brain tissues in model group, indicating a degradation of tight junction integrity in microvessels. In contrast, significant increase of claudin-5, occludin, and ZO-1 mRNA transcriptions was found in DHI group, t-PA alone treatment and combination group (**Figures 6A–C**). Compared with t-PA group, the claudin-5, ZO-1 signals were strengthened in the combination of t-PA plus DHI. Western blot analysis showed weak MMP-2 signals and strong occlusion signals (**Figure 7**). After stroke, significant increasement of the MMP-2 protein and reduction of the occluding protein were found in vehicle group. Compared with the vehicle group, the signal of MMP-2 was reduced in the combination group of t-PA plus DHI, while the signal of occludin was strengthened in the combination group.

To further evaluate the blood–brain barrier permeability of the combination group at 24 h after stroke, Evans Blue extravasation in the ischemic hemi sphere was quantified by fluorometry. After cerebral ischemia, rats had substantially more Evans Blue extravasate. Each treated group led to a pronounced decrease of postischemic BBB permeability after thrombolysis. The combination of t-PA plus DHI significantly lowered Evans Blue extravasate compared with t-PA alone treatment (**Figure 6D** and **Supplementary Figure S1**). These data showed that the combination group improved blood – brain barrier permeability.

## Discussion

Clinical studies have demonstrated that t-PA therapy is the fundamental method of treating ischemic stroke (Zhang et al., 2015). It can quickly and directly reopen occluded vessels and restore blood flow by lysis of the thrombus (Fan et al., 2010). However, besides thrombolysis *per se*, emerging data showed that exogenous t-PA may have some other pleiotropic actions in brain (Kaur et al., 2004). These actions of t-PA may increase ischemic neurotoxicity, disrupt the blood brain barrier, aggravate brain edema and hemorrhage (Kaur et al., 2004), which compromise its usefulness as a thrombolytic drug (Yepes et al., 2009; Armstead et al., 2011). So how to extend the t-PA treatment time window and reduce the risk of bleeding deserves further investigation? Reducing the dose of t-PA may overcome the dose-dependent side effects such as cerebral hemorrhage, cerebral edema and neurotoxicity, but at the same time, it would be likely to lower perfusion effect (Fan et al., 2010). Therefore, optimization of t-PA thrombolytic efficiency may require one balance into account that is thrombolytic efficiency and t-PA complication. This balance can reduce t-PA complications such as neurotoxicity, cerebral edema, and cerebral hemorrhage while ensures thrombolytic efficiency. Clearly, it prolongs the time-to-treatment window, making t-PA safer and more effective (Marder and Stewart, 2002; Dávalos, 2005).

Drug combination is a research hotspot to resolve this contradiction. The combination therapy of minocycline plus t-PA significantly reduced brain infarction, intracerebral hemorrhage and hemispheric swelling at 24 h after stroke (Fan et al., 2013). The combined application of Annexin A2 and t-PA decreased the effective thrombolytic dose of tPA, reduced hemorrhage and brain infarction, and prolonged the reperfusion time window for stroke (Zhu et al., 2010; Jiang et al., 2015). Traditional Chinese medicine (TCM) is focused on this multitargets therapy. TCM has been used in ancient medical systems for treating various neurological diseases, especially stroke, and has exerted its distinctive neuroprotective effects on cerebral ischemia (Guo et al., 2012; Liu et al., 2013). Danhong injection is widely used in traditional Chinese preparation to treat cardiovascular and cerebrovascular diseases (Du et al., 2011; He et al., 2012). We previously have demonstrated the protective effect of DHI on cerebral ischemia reperfusion injury via anticoagulant, antithrombotic, antifibrinolytic, antiinflammatory and antioxidant activities (He et al., 2012). Based on the above discussion, we speculated DHI combined with t-PA may lessen ischemic brain damage, reduced risk of intracerebral hemorrhagic transformation and lengthened the treatment window of t-PA stroke therapy.

Our study provided three major researches. (1) The optimal dose of t-PA and Danhong injection was explored for Focal Embolic Stroke. The therapeutic effects of DHI plus t-PA were systematically evaluated by neurological function evaluation, infarct volume assessment, and cerebral edema assessment. The combination of t-PA plus DHI could significantly improve neurological function, cerebral infarction, and cerebral edema in ischemia rats. The result indicated that combination thrombolytic therapy of t-PA (2.5 mg/kg) plus DHI (4 mL/kg) was more effective and specific for fibrinolysis, which was selected as a combined group for subsequent research. (2) We explored whether combination of t-PA plus DHI could extended the thrombolytic treatment time window after stroke. Rats were treated at different time after stroke. By assessment of neurological function deficits, infarct volume, cerebral edema, cerebral hemorrhage and expression of apoptotic gene, we found that combination of t-PA (2.5 mg/kg) plus DHI (4 mL/kg) which treated at 4.5 h after stroke significantly improved neurological function recovery, reduced infarct volumes, cerebral edema, inhibited the expression of proapoptotic genes, and enhanced that of inhibiting-apoptotic genes as compared the other combination groups of delayed treatment. Thus, we speculated DHI extended the t-PA treatment time windows to 4.5 h. (3) The study of protective blood–brain barrier was explored after stroke. Experimental animal studies have suggested that the disruption of blood–brain barrier may be the major contributor to increase hemorrhagic transformation and worse the neurological outcomes after stroke (Fan et al., 2017). In this study, we examined commonly used biomarkers for cerebrovascular damage, i.e., degradation of claudin-5, occludin and ZO-1, keys integral membrane proteins and components of tight junction strands. The tPA-DHI combination significantly reduced degradation of claudin-5, occludin and ZO-1 at 24 h after stroke. After cerebral ischemia, inflammatory cytokines or oxygen free radicals increased, the expression of MMPs activated, which leaded to the degradation of blood–brain barrier basement membrane and extracellular matrix. It will lead to the destruction of the blood brain barrier, aggravate cerebral edema and cerebral infarction, thus forming a vicious circle (Wang et al., 2008; Gu et al., 2012). MMP-9, and MMP-2 played the most prominent role in cerebral ischemia (Elgebaly et al., 2010; Alluri et al., 2015).

Then, MMP-9 and MMP-2 activities were tested after stroke. Results from the present study showed that the increment of plasma MMP-9 and brain MMP-2 activity after stroke were significantly inhibited by the combination of DHI with t-PA. Experimental results from this study showed that DHI (4 mL/kg) plus t-PA (2.5 mg/kg) combination therapy significantly reduced brain infarct volume, hemorrhage and brain edema at 24 h after stroke. Furthermore, our initial findings also suggest that these beneficial effects of DHI plus t-PA may be mediated by the amelioration of MMP, Evans blue higher permeability, and tight junction degradation.

The present study also has its limitations. On the one hand, we only examined the treatment of t-PA combined with Danhong injection by detecting the cerebral infarction and nervous system at 24 h after stroke. Acute neuroprotection may not always correspond to improvements of long-term outcomes. For animal experiments and clinical applications, further studies are needed to determine whether the protective effect of t-PA-DHI on nerve and the blood–brain barrier in the acute phase would be sustained. Further studies are required to confirm that t-PA combined with DHI truly correlate with improved long-term neurological outcomes, and how it interfaces with t-PA effects in extending treatment time windows after stroke. On the other hand, the underlying molecular mechanism of the combination therapy in this study is unclear, and further studies are necessary to elucidate this issue. It is hypothesized that the combination group may reduce t-PA-associated brain edema, cerebral hemorrhage and blood–brain barrier damage by reducing the dose of t-PA. Moreover, DHI has the function of improving blood flow and promoting thrombolysis. Therefore, Danhong injection combined with low-dose t-PA could improve cerebral ischemia and nerve injury, prolong t-PA treatment time window, and thereby, reduce the cerebral hemorrhage risk and neurotoxicity of t-PA. However, further investigations are needed to dissect and potentially optimize on the molecular mechanism of the combination therapy.

Our experimental results suggest that DHI (4 mL/kg) combined with t-PA (2.5 mg/kg) may reduce ischemic brain damage, ICH and blood brain barrier damage. We believe this combination of t-PA (2.5 mg/kg) plus DHI (4 mL/kg) may be ultimately facilitate new therapeutic approaches to enhance t-PA thrombolysis in stroke patients.

## Author Contributions

YZ conceived the idea and designed the study. ML and JZ participated in the experimental study and wrote the manuscript. WJ participated in the data collecting and statistical analysis. XL revised the manuscript.

## Conflict of Interest Statement

The authors declare that the researchwas conducted in the absence of any commercial or financial relationships that could be construed as a potential conflict of interest.

## Supplementary Material

FIGURE S1 The images of the evans blue infiltration after 1 day of different groups.

## References

[B1] Alluri H. Wiggins-Dohlvik K. Davis M. L. Huang J. H. Tharakan B. (2015). Blood-brain barrier dysfunction following traumatic brain injury. *Metab. Brain Dis.* 30 1093–1104. 10.1007/s11011-015-9651-7 25624154

[B2] Armstead W. M. Ganguly K. Riley J. Kiessling J. W. Cines D. B. Higazi A. A. (2011). Red blood cell-coupled tissue plasminogen activator prevents impairment of cerebral vasodilatory responses through inhibition of c-Jun-N-terminal kinase and potentiation of p38 mitogen-activated protein kinase after cerebral photothrombosis in the newborn. *Pediatr. Crit. Care Med.* 12 e369–e375. 10.1097/PCC.0b013e3181fe40a7 21037505 PMC3681424

[B3] Bambauer K. Z. Johnston S. C. Bambauer D. E. Zivin J. A. (2006). Reasons why few patients with acute stroke receive tissue plasminogen activator. *Arch. Neurol.* 63 661–664. 10.1001/archneur.63.5.661 16682535

[B4] Bederson J. B. Pitts L. H. Tsuji M. Nishimura M. C. Davis R. L. Bartkowski H. (1986). Rat middle cerebral artery occlusion: evaluation of the model and development of a neurologic examination. *Stroke* 17 472–476. 10.1161/01.STR.17.3.472 3715945

[B5] Dávalos A. (2005). Thrombolysis in acute ischemic stroke: successes, failures, and new hopes. *Cerebrovasc. Dis.* 20 135–139. 10.1159/000089367 16327264

[B6] Du J. Yang W. Yi D. Xie Y. Yang W. Zhuang Y. (2011). Analysis of using Danhong injection to treatment coronary heart disease patients medicines based on real world HIS database. *Zhongguo Zhong Yao Za Zhi* 36 2821–2824. 22292375

[B7] Elgebaly M. M. Prakash R. Li W. Ogbi S. Johnson M. H. Mezzetti E. M. (2010). Vascular protection in diabetic stroke: role of matrix metalloprotease-dependent vascular remodeling. *J. Cereb. Blood Flow Metab.* 30 1928–1938. 10.1038/jcbfm.2010.120 20664613 PMC3002883

[B8] Fan X. Jiang Y. Yu Z. Liu Q. Guo S. Sun X. (2017). Annexin A2 plus low-dose tissue plasminogen activator combination attenuates cerebrovascular dysfunction after focal embolic stroke of rats. *Transl. Stroke Res.* 8 549–559. 10.1007/s12975-017-0542-6 28580536

[B9] Fan X. Lo E. H. Wang X. (2013). Effects of minocycline plus tPA combination therapy after focal embolic stroke in type 1 diabetic rats. *Stroke* 44 745–752. 10.1161/STROKEAHA.111.000309 23422086 PMC3632085

[B10] Fan X. Qiu J. Yu Z. Dai H. Singhal A. B. Lo E. H. (2012). A rat model of studying tissue-type plasminogen activator thrombolysis in ischemic stroke with diabetes. *Stroke* 43 567–570. 10.1161/STROKEAHA.111.635250 22052516 PMC3508716

[B11] Fan X. Yu Z. Liu J. Liu N. Hajjar K. A. Furie K. L. (2010). Annexin A2: a tPA amplifier for thrombolytic stroke therapy. *Stroke* 41(10 Suppl) S54–S58. 10.1161/STROKEAHA.110.596106 20876506 PMC2994255

[B12] Gao X. Zheng X. Li Z. Zhou Y. Sun H. Zhang L. (2011). Metabonomic study on chronic unpredictable mild stress and intervention effects of Xiaoyaosan in rats using gas chromatography coupled with mass spectrometry. *J. Ethnopharmacol.* 137 690–699. 10.1016/j.jep.2011.06.024 21718771

[B13] Garcia J. H. Wagner S. Liu K. F. Hu X. J. (1995). Neurological deficit and extent of neuronal necrosis attributable to middle cerebral artery occlusion in rats. Statistical validation. *Stroke* 26 627–634. 10.1161/01.STR.26.4.627 7709410

[B14] Gu Y. Zheng G. Xu M. Li Y. Chen X. Zhu W. (2012). Caveolin-1 regulates nitric oxide-mediated matrix metalloproteinases activity and blood–brain barrier permeability in focal cerebral ischemia and reperfusion injury. *J. Neurochem.* 120 147–156. 10.1111/j.1471-4159.2011.07542.x 22007835

[B15] Guo H. Li M. J. Liu Q. Q. Guo L. L. Ma M. M. Wang S. X. (2014). Danhong injection attenuates ischemia/reperfusion-induced brain damage which is associating with Nrf2 levels *in vivo* and *in vitro*. *Neurochem. Res.* 39 1817–1824. 10.1007/s11064-014-1384-1 25069640

[B16] Guo R. B. Wang G. F. Zhao A. P. Gu J. Sun X. L. Hu G. (2012). Paeoniflorin protects against ischemia-induced brain damages in rats via inhibiting MAPKs/NF-κB-mediated inflammatory responses. *PLoS One* 7:e49701. 10.1371/journal.pone.0049701 23166749 PMC3498223

[B17] He Y. Wan H. Du Y. Bie X. Zhao T. Fu W. (2012). Protective effect of Danhong injection on cerebral ischemia-reperfusion injury in rats. *J. Ethnopharmacol.* 144 387–394. 10.1016/j.jep.2012.09.025 23010366

[B18] Henninger N. Bouley J. Bråtane B. T. Bastan B. Shea M. Fisher M. (2009). Laser Doppler flowmetry predicts occlusion but not tPA-mediated reperfusion success after rat embolic stroke. *Exp. Neurol.* 215 290–297. 10.1016/j.expneurol.2008.10.013 19038254

[B19] Jiang Y. Fan X. Yu Z. Liao Z. Wang X. S. Leyen K. V. (2015). Combination low-dose tissue-type plasminogen activator plus annexin A2 for improving thrombolytic stroke therapy. *Front. Cell. Neurosci.* 9:397. 10.3389/fncel.2015.00397 26528130 PMC4604305

[B20] Jung J. Y. Lee H. S. Kang D. G. Kim N. S. Cha M. H. Bang O. S. (2011). 1H-NMR-based metabolomics study of cerebral infarction. *Stroke* 42 1282–1288. 10.1161/STROKEAHA.110.598789 21474802

[B21] Kaur J. Zhao Z. Klein G. M. Lo E. H. Buchan A. M. (2004). The neurotoxicity of tissue plasminogen activator? *J. Cereb. Blood Flow Metab.* 24 945–963. 10.1097/01.WCB.0000137868.50767.E8 15356416

[B22] Lapchak P. A. Zhang J. H. Noble-Haeusslein L. J. (2013). RIGOR guidelines: escalating STAIR and STEPS for effective translational research. *Transl. Stroke Res.* 4 279–285. 10.1007/s12975-012-0209-2 23658596 PMC3644408

[B23] Liu Y. Tang G. H. Sun Y. H. Lin X. J. Wei C. Yang G. Y. (2013). The protective role of Tongxinluo on blood-brain barrier after ischemia-reperfusion brain injury. *J. Ethnopharmacol.* 148 632–639. 10.1016/j.jep.2013.05.018 23707212

[B24] Marder V. J. Stewart D. (2002). Towards safer thrombolytic therapy^∗^. *Semin. Hematol.* 39 206–216. 10.1053/shem.2002.34088 12124683

[B25] Ploen R. Sun L. Zhou W. Heitmeier S. Zorn M. Jenetzky E. (2014). Rivaroxaban does not increase hemorrhage after thrombolysis in experimental ischemic stroke. *J. Cereb. Blood Flow Metab.* 34 495–501. 10.1038/jcbfm.2013.226 24346690 PMC3948130

[B26] Wang X. Rosell A. Lo E. H. (2008). Targeting extracellular matrix proteolysis for hemorrhagic complications of tPA stroke therapy. *CNS Neurol. Disord. Drug Targets* 7 235–242. 10.2174/187152708784936635 18673208

[B27] Wang Z. Song F. Li J. Zhang Y. He Y. Yang J. (2014). PET demonstrates functional recovery after treatment by Danhong injection in a rat model of cerebral ischemic-reperfusion injury. *Evid. Based complement. Alternat. Med.* 2014:430757. 10.1155/2014/430757 24707308 PMC3953511

[B28] Weintraub M. I. (2006). Thrombolysis (tissue plasminogen activator) in stroke: a medicolegal quagmire. *Stroke* 37 1917–1922. 10.1161/01.STR.0000226651.04862.da 16728683

[B29] Whiteley W. N. Thompson D. Sandercock P. (2014). Response to letter regarding article, “Targeting recombinant tissue-type plasminogen activator in acute ischemic stroke based on risk of intracranial hemorrhage or poor functional outcome: an analysis of the Third International Stroke Trial”. *Stroke* 45:e133. 10.1161/STROKEAHA.114.005871 24916910 PMC4282174

[B30] Yepes M. Roussel B. D. Ali C. Vivien D. (2009). Tissue-type plasminogen activator in the ischemic brain: more than a thrombolytic. *Trends Neurosci.* 32 48–55. 10.1016/j.tins.2008.09.006 18963068

[B31] Zhang L. Zhang R. L. Jiang Q. Ding G. Chopp M. Zhang Z. G. (2015). Focal embolic cerebral ischemia in the rat. *Nat. Protoc.* 10 539–547. 10.1038/nprot.2015.036 25741989 PMC4602402

[B32] Zhi X. W. Su X. M. Feng W. Y. Zhang H. M. (2012). Effect and mechanism of Danhong injection on isolated mesenteric arterial rings in rats. *Zhongguo Zhong Yao Za Zhi* 37 2607–2611. 23236761

[B33] Zhu H. Fan X. Yu Z. Liu J. Murata Y. Lu J. (2010). Annexin A2 combined with low-dose tPA improves thrombolytic therapy in a rat model of focal embolic stroke. *J. Cereb. Blood Flow Meta.* 30 1137–1146. 10.1038/jcbfm.2009.279 20068577 PMC2949213

