# Supplementary figures and images for: Danhong Injection Combined With t-PA Improves Thrombolytic Therapy in Focal Embolic Stroke
Source: Front Pharmacol. 2018 Apr 6;9:308. doi: 10.3389/fphar.2018.00308 (PMC5897498; doi:10.3389/fphar.2018.00308)

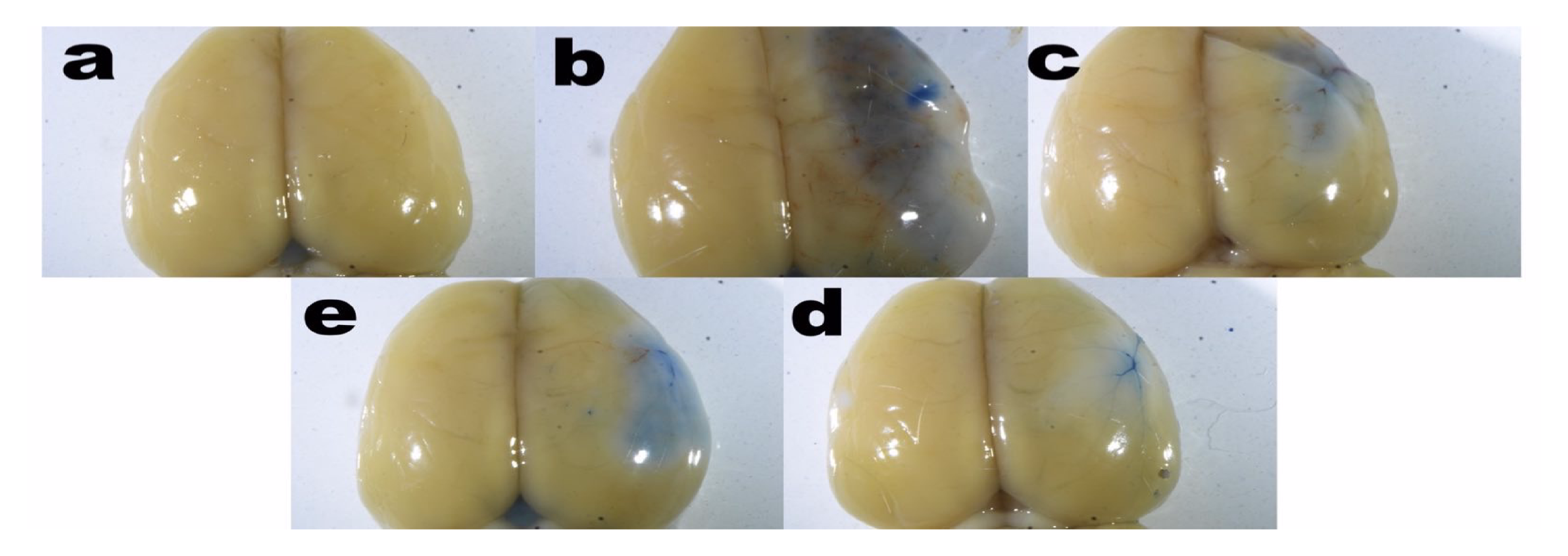

Supplement: FIGURE S1 — The images of the evans blue infiltration after 1 day of different groups. [file Image_1.TIF]
